# Aspiration pneumonia and death in Huntington’s disease

**DOI:** 10.1371/currents.RRN1293

**Published:** 2012-02-02

**Authors:** Anne-Wil Heemskerk, Raymund A.C. Roos

**Affiliations:** ^*^Researcher and Speech and Language Pathologist (SLP) at LUMC, dept. Neurology, Leiden, The Netherlands and ^†^LUMC, Leiden, The Netherlands

## Abstract

Huntington’s disease (HD) is a progressive neurodegenerative autosomal dominant disease characterized by choreatic and hypokinetic movements, disturbed behaviour, and cognitive decline. Pneumonia is the most common cause of death, followed by cardiovasculair diseases. It has been suggested that choking is the causative underlying factor for pneumonia in HD. As a detailed specification of the type of pneumonia has never been performed, we analyzed the records of our Brain Bank containing 224 cases to determine the exact cause of death and type of pneumonia. The conclusion is that the majority (86.8%) of our HD patients where the cause of death could be identified died from aspiration pneumonia.

## **Introduction**

Huntington’s disease (HD) is a progressive neurodegenerative autosomal dominant disease characterized by disturbed movements, changes in behaviour, and cognitive decline. Although the motor disturbances are both choreatic and hypokinetic, chorea is the most characterizing. Choreatic movements are irregular, unwanted involving not only the limbs and the trunk but also the respiratory and buccolingual muscles. HD is caused by a CAG repeat expansion of the HTT gene on the short arm of chromosome 4. The mutant protein huntingtin causes neurodegeneration in the brain, particularly in the caudate nucleus and putamen. The mean age at onset is in the third and fourth decade of life and the disease duration about 15-20 years. [1] Death usually results from respiratory complications. Studies to the cause of death in HD found that the primary cause (variation of 33%-85.7%.) of death is pneumonia. [2] [3] [4] [5] [6] [7] None of the reported studies mentioned any details about the type of pneumonia. Pneumonia has several causative factors of which aspiration seems an important one. Pneumonia can be classified into different types, such as community-acquired pneumonia (CAP), hospital-acquired pneumonia (HAP), chemical pneumonia, and aspiration pneumonia. Although the exact percentage is unknown, most HD patients have dysphagia, especially in the advanced stage of the disease, therefore, aspiration is the most likely cause of the fatal pneumonia. The aim of the present study is to find out how often aspiration pneumonia is the primary type of fatal pneumonia in HD. 

## **Materials and Methods**

The records of all deceased HD patients from the Leiden University Medical Center (LUMC) brainbank in the Netherlands were collected. The diagnosis of HD was confirmed by family history and post-mortem pathological investigation, and since 1993 in most cases by DNA analysis. From all files the following information was collected: DNA confirmation, gender, year of birth, year of death, age at onset, location of death, naturally death, total body autopsy, described primary cause of death, other described underlying causes of death, repeated pneumonias, special remarks. The special remarks contained all available information about the type of pneumonia and the macroscopic description of the content of the lungs at autopsy. The clinical data from the patients were analyzed, when available, especially for dysphagia. 

### Statistical analysis

Descriptive statistics was used to obtain results, and Chi-square distribution was used to compare different variables. (SPSS 18.0).   

## **Results**

Two hundred twenty four charts of deceased HD patients were reviewed, of which 216 (111/104 male/female, one unknown) had records available, 147 listed a cause of death (Table 1), which was pneumonia in 81 (55%) cases. 38 of those 81 cases had an autopsy that included a description of the lungs. Of those 38 cases, 12 had gastric juices or food in the lungs, and 4 had giant cells, reaction which we defined as “death due to aspiration pneumonia”, 17/38 had hyperemia, which we defined as “possible aspiration pneumonia”, and only 5 showed bacteria or viruses, which was defined as “primary infectious pneumonia”. For the cause of death aspiration pneumonia, no significant difference was found for gender and aspiration (χ^2^=.995). From all 216 records, in 69 cases no primary cause of death was given. In 15/69 records the clinical information revealed information about dysphagia, and in 8/69 records information about repeated pneumonias in the end stage was present. In 4/69 records, a description of the microscopy revealed of giant cells and hyperemia in the lungs.   

**Table:** 

**Cause of death**	**N=147**	**100%**
Known cause: - pneumonia - suffocation - pulmonary embolism - cachexia - cardiac diseases - other neurological diseases - shock/sepsis - suicide - euthanasia - other causes	81 6 6 11 16 3 7 2 5 10	55.1 4.1 4.1 7.5 10.9 2.0 4.8 1.4 3.4 6.8

Table 1: Primary cause of death in 147 patients with Huntington disease

## **Discussion**

With the information available, we found it plausible that significant more patients died from aspiration pneumonia, instead of a primary infectious pneumonia. It is most likely that dysphagia is the causative factor for this aspiration pneumonia. The relation between dysphagia leading to aspiration pneumonia is confirmed in patients with Parkinson disease (PD) and in elderly people. [8] [9] The leading cause of death in PD is aspiration pneumonia, for as much as 70%. And these patients also suffer from dysphagia. [8] [10] [11] In elderly people it is found that dysphagia is a common problem, and that dysphagia is the major pathophysiologic mechanism leading to aspiration pneumonia.[9] [12] Another interesting point is that it is likely that patients who died from an infectious pneumonia, still died because of aspiration. Because in most cases the cultures consisted of the staphylococcus aureus, klebsiella and candida albicans. When patients aspirate their saliva, patients can develop pneumonia, especially patients in the last stage of HD, who are mostly cachectic, in bad condition and with a poor resistance. Previous studies to the role of chronic conditions, health behaviors, and nutritional status have shown that these factors increases the risk of pneumonia. [13] [14] [15] On investigating the files of the deceased HD patients, a large proportion of the files, 32% did not contain the primary cause of death. Therefore, it seems that the files of the deceased HD patients are relatively inaccurate in recording causes of death. Other studies have also shown an inaccuracy rate. Haines and Conneally [4] had an overall rate of 66%. Alderson [16] found an overall accuracy rate of 61%. Thus, our overall rate of 68.1% is not unusual. Because of this overall rate, it seems likely that more patients died of aspiration pneumonia. Some of these patients were described with suffering from dysphagia, and repeated pneumonias in their last period of life. As said these issues have influence on developing aspiration pneumonia. To conclude, our data suggest that aspiration pneumonia is the most prominent primary cause of death in HD, which is in accordance with the literature. As the source data showed to be rather incomplete, lacking information about the clinical status of the patients regarding dysphagia, we started to develop a dysphagia assessment scale to get better insight in the prevalence of dysphagia and its consequences for the development of pneumonia.

## **Competing interests**

The authors have declared that no competing interests exist.

## **Funding information**

This study is funded by the Jacques and Gloria Gossweiler Foundation, a non-profit organization under the Swiss law.
